# Low-dose decitabine enhances the effect of PD-1 blockade in colorectal cancer with microsatellite stability by re-modulating the tumor microenvironment

**DOI:** 10.1038/s41423-018-0026-y

**Published:** 2018-04-05

**Authors:** Ganjun Yu, Yanfeng Wu, Wenying Wang, Jia Xu, Xiaoping Lv, Xuetao Cao, Tao Wan

**Affiliations:** National Key Laboratory of Medical Immunology & Institute of Immunology, Second Military Medical University, 800 Xiangyin Road, Shanghai, 200433 China

**Keywords:** colorectal cancer, decitabine, microsatellite stability, PD-1 blockade, tumor microenvironment

## Abstract

PD-1 blockade has demonstrated impressive clinical outcomes in colorectal cancers that have high microsatellite instability. However, the therapeutic efficacy for patients with tumors with low microsatellite instability or stable microsatellites needs further improvement. Here, we have demonstrated that low-dose decitabine could increase the expression of immune-related genes such as major histocompatibility complex genes and cytokine-related genes as well as the number of lymphocytes at the tumor site in CT26 colorectal cancer-bearing mice. A more significant inhibition of tumor growth and a prolongation of survival were observed in the CT26 mouse model after treatment with a combination of PD-1 blockade and decitabine than in mice treated with decitabine or PD-1 blockade alone. The anti-tumor effect of the PD-1 blockade was enhanced by low-dose decitabine. The results of RNA sequencing and whole-genome bisulfite sequencing of decitabine-treated CT26 cells and tumor samples with microsatellite stability from the patient tumor-derived xenograft model have shown that many immune-related genes, including antigen-processing and antigen-presenting genes, were upregulated, whereas the promoter demethylation was downregulated after decitabine exposure. Therefore, decitabine-based tumor microenvironment re-modulation could improve the effect of the PD-1 blockade. The application of decitabine in PD-1 blockade-based immunotherapy may elicit more potent immune responses, which can provide clinical benefits to the colorectal cancer patients with low microsatellite instability or stable microsatellites.

## Introduction

In recent years, tumor immunotherapy has attracted increasing amounts of attention. Among these approaches, PD-1 blockade has shown exciting clinical outcomes in melanoma, non-small-cell lung cancer, bladder cancer, Hodgkin lymphoma and other solid tumors.^1–7^ Although PD-1 blockade, which is representative of checkpoint inhibitors, has become a hot topic in the tumor immunotherapy field, its clinical effectiveness is still limited: the overall objective response rate is only 20–30%.^8,9^ For colorectal cancer (CRC), PD-1 blockade was not as effective as previously thought.^7,10^ However, a group from Johns Hopkins Sidney Kimmel Comprehensive Cancer Center recently reported that CRC with the characteristic of high microsatellite instability (MSI-H) was sensitive to PD-1 blockade therapy.^11^ Later, in May 2017, the US Food and Drug Administration approved Keytruda (a PD-1 blockade agent) for the treatment of patients with unresectable or metastatic solid tumors that have been identified as MSI-H or mismatch repair (MMR) deficient.

As early as the 1980s, researchers found that a deficiency of the MMR (dMMR) in CRC frequently resulted in MSI-H, which was closely related to the prognosis.^12^ Subsequently, more researchers reported that in MSI-H/dMMR CRC, the abnormal protein expression that resulted from DNA mutations or deletions could not be effectively corrected.^13,14^ These abnormal proteins would accumulate and then be processed and presented as abnormal epitopes and become so-called “neoantigens”.^15^ These neoantigens were presumed to be strongly immunogenic and able to recruit dendritic cells, T lymphocytes and other immune cells to infiltrate the tumor site and potentially break the original inhibitory status of the tumor microenvironment (TME) and then trigger a new specific immune response.^16^ Unfortunately, these activated immune cells would rapidly become anergic because of the inducible PD-L1 expressed by the tumor cells. This effect explains the fact that the patients with MSI-H/dMMR had good clinical response to PD-1 blockade. However, only ~15% of all CRC patients exhibit MSI-H,^17,18^ which implies that the majority of CRC patients would not benefit from PD-1 blockade. Therefore, it has become urgent to determine methods to improve the clinical response and survival of patients with tumors that express low microsatellite instability (MSI-L) or that are microsatellite stable (MSS).

Because of the rarity of the neoantigens, improving the tumor-associated antigen (TAA) expression and immunogenicity of tumor cells may be a good option. We know that the methylation level of promotor regions is tightly related to the “open-close” state of genes.^19^ With the recent progress in epigenetics research, the demethylation effect of decitabine (5-aza-2′-deoxynucleoside, DAC) has been increasingly noted, and DAC has been widely used as a chemotherapy drug in myelodysplastic syndrome and leukemia.^20–22^ A specific DNA methyltransferase inhibitor, DAC is a cytosine analog that reverses DNA methylation and induces tumor cell differentiation or apoptosis.^23,24^ A low dose of DAC can replace cytosine in tumor cells, where it covalently binds to DNA methyltransferase, which results in the degradation of this enzyme but does not terminate the cell cycle.^25^ In summary, low-dose DAC reactivates the silenced genes by demethylation of the promoter region. It is worth mentioning that low-dose DAC can not only significantly improve the expression of NY-ESO-1, MAGE-A3/6, which are important TAAs commonly used as targets for tumor immunotherapy, but also remarkably upregulate the expression of co-stimulatory molecules, major histocompatibility complex (MHC) class I and other immune markers on tumor cells.^26,27^ These results indicate that low-dose DAC can improve the expression of TAAs and modify the tumor cells to be easily recognized by immune system.

Due to the ineffectiveness of the PD-1 blockade in MSI-L or MSS patients and the role of low-dose DAC, low-dose DAC may be a good pretreatment adjunct to PD-1 blockade. Here, we have investigated the modification of CRC cells by DAC, the mechanism of DAC’s effects of improving the immune-related gene expression in tumor cells and a patient tumor-derived xenograft (PDX) model and re-modulating the TME in a mouse model of CRC. More importantly, we also examined whether DAC-mediated TME re-modulation could improve the response to PD-1 blockade, which may benefit those CRC patients with MSI-L or MSS tumors.

## Materials and methods

### Cell line and DAC treatment

The mouse CRC cell line CT26 was preserved by our laboratory and cultured in RPMI-1640 medium (PAN, Wimborne, UK) supplemented with 10% fetal bovine serum (Biowest-Uruguay, Nuaillé, France). Decitabine (DAC) was purchased from Janssen Pharmaceutical Ltd., Xian. An anti-PD-1 antibody (clone: J43) was kindly provided by Prof. Lianjun Shen from Hengrui Pharmaceutical Co. Ltd., Shanghai. Cytidine (Sigma, MO, USA) was purchased from Univ Biotech Co., Ltd. as a control drug.

CT26 cells were seeded at a density of 1 × 10^5^ cells/ml in a six-well culture plate. After 24 h, the medium was replaced with complete medium containing 1 μM DAC every 24 h for 3 days. After the third DAC treatment, the medium was replaced with fresh complete medium without DAC, and the cells were cultured for an additional 24 h and then used for subsequent experiments. Control groups were treated synchronously under the same conditions except for the absence of DAC.

### PDX model

With the approval of the ethics committee and patients’ consent, fresh tumor tissues from patients with CRC were obtained from Shanghai Changzheng Hospital, China. The MSI status was determined by PCR of genomic DNA isolated from tumor tissue and paracancer tissue from patients. Five markers (BAT-26, BAT-25, D5S346, D2S123, and D17S250) that are recommended by National Cancer Institute for the uniform analysis of MSI in colorectal cancer were evaluated.^28^ Tumor samples identified as MSS were selected for further PDX model establishment. After the non-tumor tissue and necrotic tissue were removed, the tumor tissue was cut into multiple 2 × 2 × 2 mm^3^ pieces for subcutaneous grafting under the right axillary site of the 6-week-old to 8-week-old female NOD-Prkdc^em26Cd52^Il2rg^em26Cd22^/NjuCrl mice (Nanjing Galaxy Biopharma Co., Ltd, Nanjing, China). At 28 days post-inoculation, we administered DAC 20 μg to each mouse for 5 consecutive days by intraperitoneal injection. The control groups were given the same volumes of saline in the same manner. On the seventh day after the end of the drug treatment, the tumors were collected and preserved in RNAlater stabilization solution (Thermo Fisher, CA, USA) for future use.

### Whole-Genome Bisulfite Sequencing (WGBS) and data analysis

Genomic DNA was isolated from the control and the DAC-treated CT26 cells and PDX tumors using a QIAamp genomic DNA Kit (Qiagen, Stockach, Germany) according to the instructions. Bisulfite treatment of genomic DNA was performed using a DNA Methylation Lightning Kit (Zymo EZ, CA, USA). Briefly, 200–500 ng of purified genomic DNA was treated with the Zymo Lightning Conversion Reagent in a thermal cycler for 8 min at 98 °C, followed by 60 min at 54 °C. The bisulfite-treated DNA was purified on a spin column and used to prepare the sequencing library using an EpiGnome Kit (Epicentre, Wisconsin, USA). In this procedure, bisulfite-treated single-stranded DNA was random-primed using a polymerase able to read uracil nucleotides to synthesize DNA that contained a specific sequence tag. The 3′ ends of the newly synthesized DNA strands were then selectively tagged with a second specific sequence, which resulted in doubly tagged DNA molecules with known sequence tags at their 5′ and 3′ ends. The EpiGnome libraries were diluted and loaded onto the cBot DNA Cluster Generation System. After the cluster generation was completed, the flow cell was transferred to the HiSeq 3000 System for sequencing using 75 bp paired-end reads. Additional sequencing could be completed for higher coverage when necessary. The data were analyzed according to the GO database and KEGG database.

### RNA sequencing (RNA-seq) and data analysis

Sequencing and analysis were performed individually on the control and DAC-treated CT26 cells and PDX tumors. The total RNA was isolated with TRIzol (Sigma, St Louis, US) according to the manufacturer’s instructions and converted into libraries of double-stranded cDNA as a template for high-throughput sequencing using the Illumina CBot station and HiScanSQ using the Illumina TruSeq RNA Sample Preparation Kit according to the manufacturer’s recommendations. Briefly, mRNA was purified from 10 to 20 μg of total RNA using poly-T oligo-attached magnetic beads. Fragmentation was conducted using divalent cations under an elevated temperature in the proprietary Illumina fragmentation buffer. Double-stranded cDNA was constructed and subsequently used to establish the cDNA library. RNA-seq was performed on a Hiseq 3000 system. Fragments per kilobase of exon model per million mapped reads (FPKM) values for Refseq genes were established using CASAVA 1.8. To identify reads that spanned alternative splicing events or gene fusion breakpoints, we also analyzed reads using TopHat and Bowtie.

### Immunohistochemistry and evaluation

Formalin-fixed, paraffin-embedded tumor tissues were processed for immunohistochemical staining with antibodies for CD3 (Cell Signaling Technology, clone: D4V8L, Shanghai, China), CD4 (Abcam, clone: EPR19514, MA, USA), CD8 (Abcam, clone: EPR20305, MA, USA), PD-1 (R&D, MN, USA), or interferon-γ (IFN-γ) (Abcam, MA, USA). The antigen retrieval procedure for enhanced detection involved immersion of tissue sections in Tris-EDTA buffer solution (pH 9.0) and heating using microwave irradiation for 5 min. After antigen retrieval and a cooling-off period, the endogenous peroxidase activity was inactivated with 3% hydrogen peroxide in methanol for 10–15 min at 37 °C. The antibodies were incubated with the tissue sections for 2–4 h at room temperature in a humidified chamber. After incubation with primary antibodies, the immunohistochemical procedures were conducted with the ChemMate DAKO Envision Detection Kit (Peroxidase/DAB, Rabbit/Mouse, Glostrup, Denmark) according to the instructions.

Images were acquired using the PerkinElmer Vectra 3 IHC Image Processing System. Briefly, the numbers of tumor-infiltrating T lymphocytes in TME were calculated as follows: ten fields at low magnification (×20) in the tumor nests with the most infiltrating T lymphocytes were selected and then counted and analyzed using Inform 2.0 software. The average value was acquired from the results of the ten selected areas and subsequently used in the statistically analysis.

### Treatment of mice with DAC followed by PD-1 blockade

A total of ~10^6^ CT26 cells in 100 μl PBS were inoculated into the lateral flanks of each BALB/c mouse (Sippr-BK, Shanghai, China). The treatments were initiated on the seventh day after tumor inoculation. DAC was administered by intraperitoneal injection, 20 µg/mouse for 5 consecutive days. After that, PD-1 blockade was performed by injecting 500 μg of anti-PD-1 antibody into each mouse every 3 days through the tail vein for a total of 4 doses. The longest and shortest diameters (*L* and *W*) of the tumors were measured using Vernier calipers (Sata, Shanghai, China) every 3 days. The tumor volume (*V*) was calculated by the formula: *V* = (*L* × *W*^2^)/2. The survival of the tumor-bearing mice was observed and recorded every 3 days.

### Statistical analysis

The statistical analysis was performed using SPSS 21.0 software (IBM Corporation, NY, USA). Student’s *t*-test was used to compare the differences in the tumor sizes and positive cell counts between two groups. ANOVA was used for comparisons among the groups for results involving combinations of DAC with PD-1 blockade. The Kaplan–Meier survival analysis and log-rank test were used for comparisons of the survival of the mice. A *p*-value of <0.05 was considered to be a statistically significant difference.

## Results

### Demethylation of promoter region by low-dose DAC in the tumor cells

**Fig. 1:**
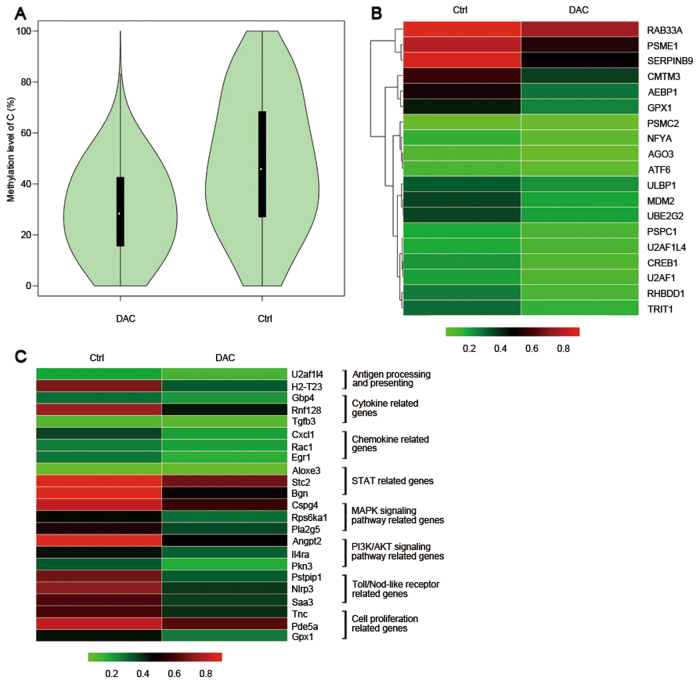
Low-dose DAC decreased the methylation level of the promoter region in CT26 cells. **a** The violin map shows the methylation levels of the control and DAC-treated CT26 cells. Each violin represents a sample, and every 10 kb was regarded as a bin. The methylation level of each bin was calculated. The wider the violin’s was, the more cytosine was methylated. The white dots in the violin represent the median, the black boxes indicate the IQR (lower quartile to upper quartile), the thin black lines indicates the whiskers, and the violin’s shape represents the density of the distribution. **b** Comparison of the methylation level of antigen-processing and antigen-presenting gene promoter regions in the control and the DAC-treated CT26 cells. **c** Comparison of the methylation level of representative differentially expressed genes of the control and DAC-treated CT26 cells

Because low-dose DAC is known to act as a methyltransferase inhibitor, we determined the methylation levels of the promoter regions of the whole genome by WGBS. After analysis, we found that the methylation level of the CT26 cells was significantly downregulated after DAC treatment (Fig. 1a). We observed that the methylation levels of the promoter regions of antigen-processing and antigen-presenting genes (Fig. 1b, c), cytokine-related genes, and chemokine-related genes were downregulated (Fig. 1c). A consistent effect was also found for STAT genes, immune response genes, MAPK-signaling pathway genes and PI3K-AKT-signaling pathway genes (Fig. 1c). Thus, low-dose DAC played a demethylation role in the treatment course.

**Fig. 2:**
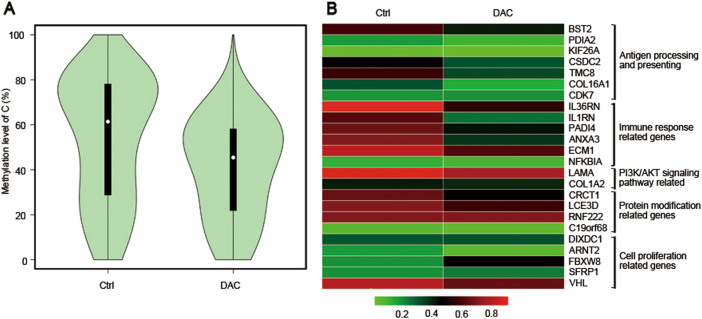
Low-dose DAC decreased the methylation level of the promoter region in tumors in the PDX model. **a** The violin map shows the methylation levels of the control and DAC-treated tumors. **b** Comparison of the methylation level of representative differentially expressed genes in the control and the DAC-treated tumors

Similar results were also found in the PDX model. The methylation level of the tumor cells was significantly downregulated after DAC treatment (Fig. 2a). Accordingly, in the DAC-treated condition, the promoter region of genes such as antigen-processing and antigen-presenting genes, immune-related genes and protein modification genes were at low demethylation level (Fig. 2b).

### TME re-modulation by the effects of DAC on tumor cells

**Fig. 3:**
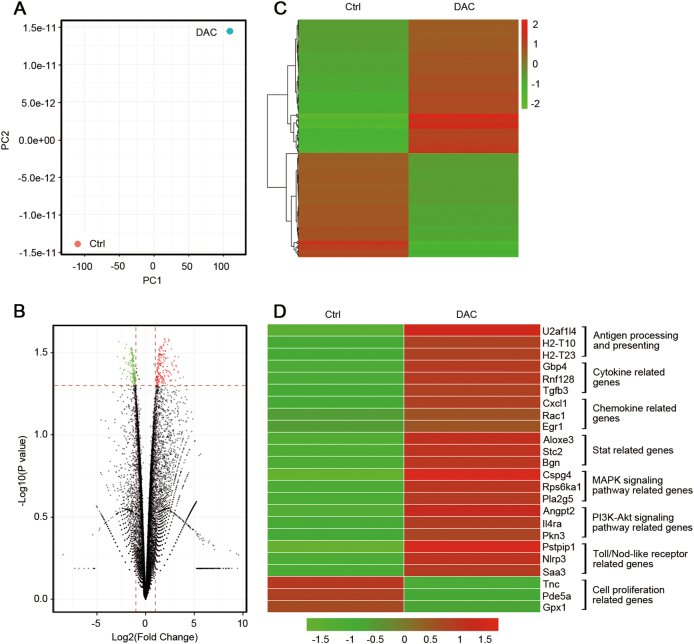
The effects of low-dose DAC on the CT26 cells. **a** Principal component analysis of the control and DAC-treated CT26 cells. A greater distance between the two points indicates a greater difference in the gene expression. **b** The volcano plots show the fold-changes (FC) and *p*-values for the comparisons between the control and DAC-treated CT26 cells. Differentially expressed genes (FC ≥ 2, *p*-value <0.05 with FDR, diffå 100) are displayed in red, and differentially expressed genes (FC ≤ 2, *p*-value <0.05 with FDR, diffå 100) in green. **c** Unsupervised hierarchical clustering of the control and DAC-treated CT26 cells. **d** Representative differentially expressed genes and their associated functions in the control and DAC-treated CT26 cells

**Fig. 4:**
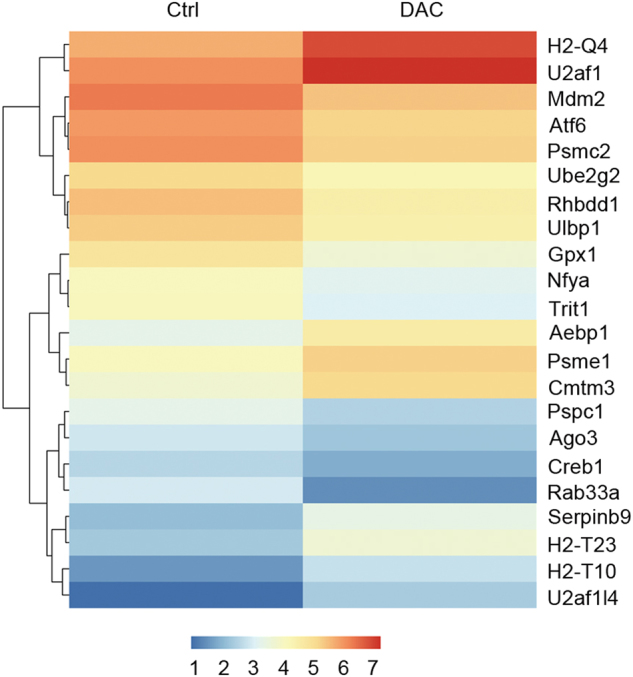
Comparison of the differentially expressed antigen-processing and antigen-presenting genes in the control and DAC-treated CT26 cells

To confirm the relationship between the methylation level of genes and their expression in the tumor cells, which play an important role in the TME, we conducted a RNA-seq analysis of the DAC-treated and untreated CT26 cells. We focused on the mRNA expression level of the genes indicated by the WGBS results. Our data suggested that the expression profile of CT26 cells was obviously changed (Fig. 3a). A number of functionally related genes were differentially expressed after DAC exposure, including more than 180 genes that were significantly upregulated (red) and 139 genes that were significantly downregulated (green) (Fig. 3b, c). We also found that antigen-processing and antigen-presenting genes, cytokine-related genes, chemokine-related genes, STAT genes, immune response genes, MAPK-signaling pathway genes and PI3K-AKT-signaling pathway genes were activated (Fig. 3d). Notably, the genes related to antigen processing and presenting were significantly upregulated (Fig. 4), which suggested that the immunogenicity of tumor cells was improved. The differentially expressed genes that were downregulated after DAC treatment were mainly related to cell proliferation (Fig. 3d).

**Fig. 5:**
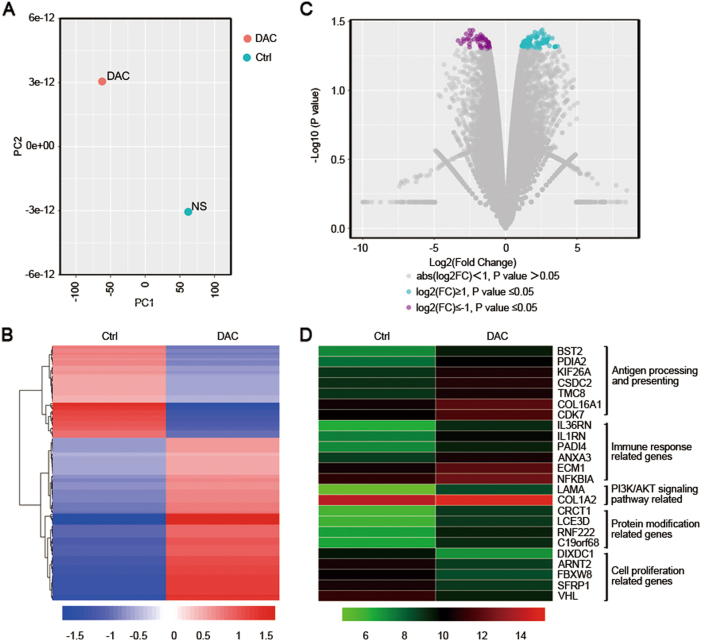
The effects of low-dose DAC in the PDX model. **a** Principal component analysis of the control and DAC-treated tumors in the PDX model. A greater distance between two points indicates a greater difference in the gene expression. **b** Unsupervised hierarchical clustering of the control and the DAC-treated tumors. **c** The volcano plots show the fold-changes (FC) and *p*-values for the comparisons between the control and the DAC-treated tumors. Differentially expressed genes (FC ≥ 2, *p*-value <0.05 with FDR, diffå 100) are displayed in purple, and differentially expressed genes (FC ≤ 2, *p*-value <0.05 with FDR, diffå 100) are displayed in green. **d** Representative differentially expressed genes and their related functions in the control and the DAC-treated tumors

Results similar to those described above were also observed in the PDX model. The expression profile of the tumors that were primarily identified as MSS (data not shown) was changed after DAC treatment in vivo (Fig. 5a, b). Approximately 89 genes were significantly upregulated (green) whereas 50 were downregulated (purple; Fig. 5c). The major types of upregulated genes were antigen-processing and antigen-presenting genes, immune-related genes and protein modification genes (Fig. 5d). These results focused on the genes with low methylation levels, as shown by WGBS. Cell proliferation-related genes were downregulated, which was consistent with the data from the CT26 cells (Fig. 5d). All the above results indicated that DAC could break the balance of the primary TME and re-modulate it to a novel status by activating certain genes via demethylation of the promoter regions.

### TME re-modulation by the influence of DAC on immune cells

**Fig. 6:**
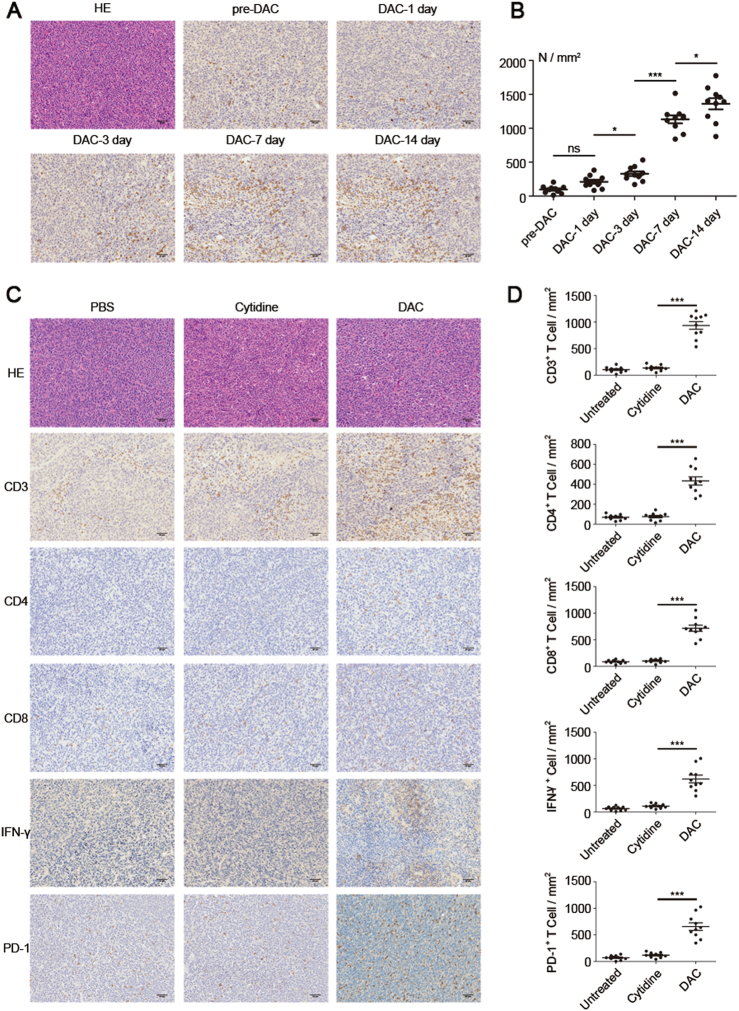
Re-modulation of the tumor microenvironment by low-dose DAC. **a** The mice were inoculated with the tumor, and beginning 7 days later, each mouse received peritoneal injections of 20 μg DAC for 5 consecutive days. The last day of injection was recorded as DAC-0 day. The mice were sacrificed at DAC-1 day, DAC-3 day, DAC-7 day and DAC-14 day. The tumor sections were stained for CD3 by IHC. **b** The stained slides were analyzed using the PerkinElmer Vectra 3 automated, high-throughput quantitative pathology imaging system. CD3-positive T cells were counted under ×200 magnification. From each slide, 10 fields were selected for analysis. The results were analyzed using ANOVA. *p < 0.05, ***p < 0.001, ns indicates no significant difference. **c** The tumors on DAC-7 day from the PBS-treated, Cytidine-treated, and DAC-treated mice were stained for CD3, CD4, CD8, IFN-γ and PD-1. **d** The stained slides were analyzed using the PerkinElmer Vectra 3 automated, high-throughput quantitative pathology imaging system. The positive cells were counted under ×200 magnification. From each slide, 10 fields were selected for analysis. The results were analyzed using ANOVA. ***p < 0.001

DAC at an adaptive dose could induce a number of functional genes in tumor cells to be differentially expressed, which resulted in TME re-modulation. We therefore wondered if DAC treatment could further modify the TME by affecting immune cells such as T lymphocytes, which are one of the most important soldiers in the anti-tumor battle. Tumor-bearing mice were treated with low-dose DAC for 5 consecutive days, and the end day was recorded as DAC-0 day. The mice were sacrificed at a series of different times as indicated in Fig. 6a, and tumors were excised. Excitingly, we found that increasing numbers of T lymphocytes infiltrated into the tumor area after the low-dose DAC treatment. The number of infiltrated T lymphocytes was significantly increased at the DAC-3 day, and reached a peak at the DAC-7 day and DAC-14 day (Fig. 6b).

To further evaluate the status of the modification of the TME by low-dose DAC, we treated the tumor-bearing mice with DAC for 5 consecutive days. Parallel controls included the use of PBS and cytidine (an analog of DAC). The tumors were isolated at Day 7 and then tested using IHC (Fig. 6c). There were many more T lymphocytes, including CD4^+^ T lymphocytes and CD8^+^ T lymphocytes in the TME, and the IFN-γ secretion level was significantly greater in the DAC-treated group than in the control groups (Fig. 6d). Notably, we found that the majority of the infiltrated T lymphocytes were PD-1 positive (Fig. 6c), which suggested that the re-modulation of the TME by DAC might maintain the inhibition status induced by the PD-1 blockade.

### Improvement of the anti-tumor effect of PD-1 blockade with DAC assistance

**Fig. 7:**
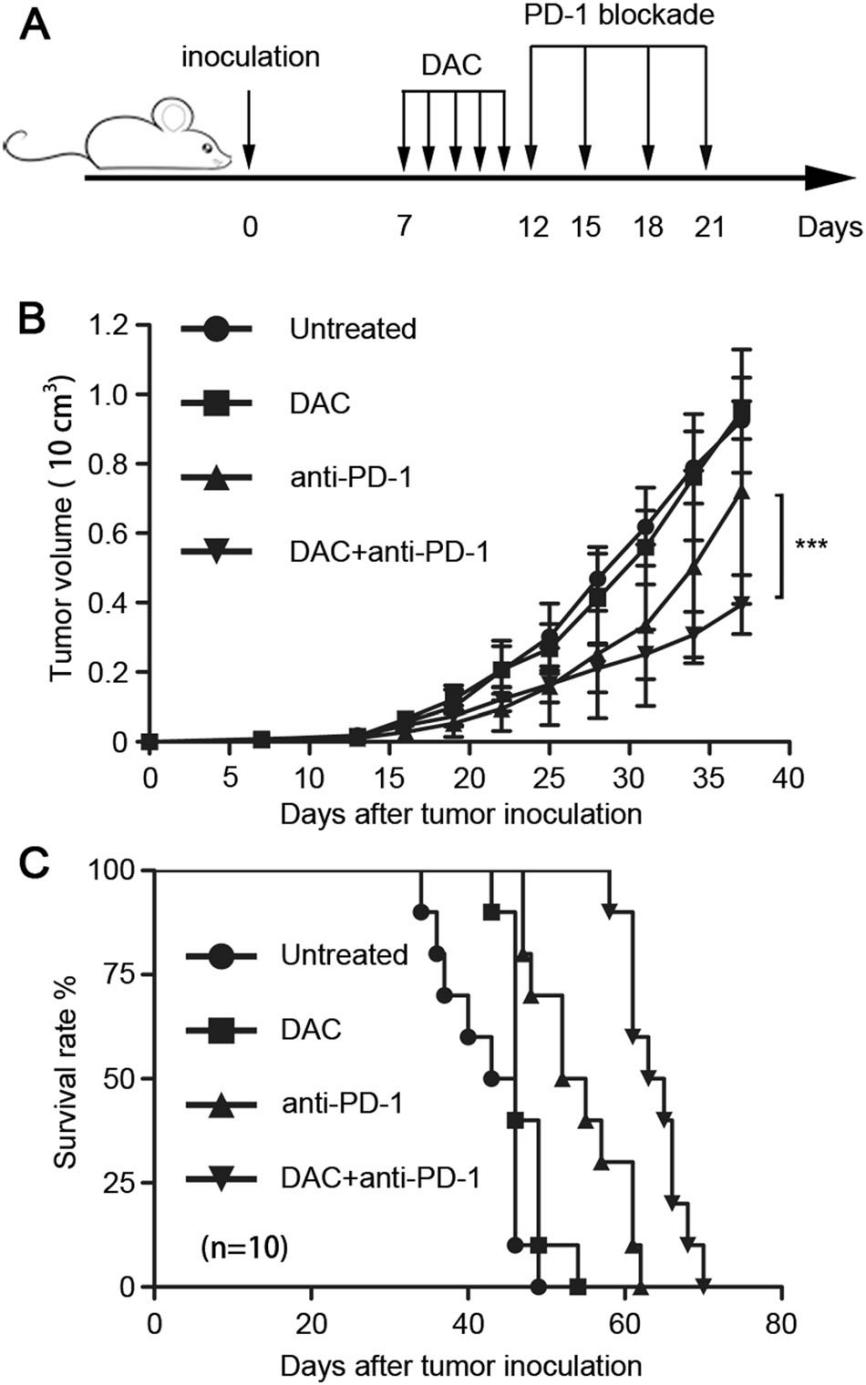
The anti-tumor effect of a PD-1 blockade was improved by low-dose DAC in CT26 tumor-bearing mouse model. **a** Seven days after the tumor inoculation, the mice received peritoneal injections of low-dose DAC (20 μg/mouse) for 5 consecutive days. Subsequently, one group was administered the PD-1 blockade (500 μg/per mouse) by tail vein injection every 3 days for a total of four doses. The mice in the other three groups were injected with the same volume of saline at the corresponding time. **b** The tumor volumes were evaluated and recorded every 3 days by measuring the longest and shortest diameters (*L* and *W*). The tumor volumes (V) were calculated using the formula: *V* = (*L* × *W*^2^)/2. The results were analyzed using ANOVA. ***p < 0.001. **c** The survival rates of the mice were observed and recorded, and the results were analyzed using the log-rank test

Since DAC could re-modulate the TME by modifying the tumor cell characteristics with the consequence of improving the T lymphocyte infiltration, we hoped to improve the therapeutic effect of PD-1 blockade by the addition of DAC. Mice bearing CT26 tumors were treated with various drug combinations (Fig. 7a). The results showed that DAC alone (*n* = 10) had no effect on tumor growth (Dunnett *t*-test, *P* = 0.060) compared to the untreated group, but the survival rate improved (log-rank test, *P* = 0.0273). PD-1 blockade alone (*n* = 10) began to show an inhibitory effect compared with the untreated group at Day 19 (Dunnett *t*-test, *P* = 0.039) and improved the survival rate (log-rank test, *P* = 0.004). The combination of DAC and PD-1 blockade (*n* = 10) showed a significantly different effect on tumor inhibition on Day 34 (Dunnett *t*-test, *P* = 0.019) and a longer survival time than the PD-1 blockade alone (log-rank test, *P* < 0.001 (Fig. 7b, c). Therefore, DAC modified the tumor cells to be more sensitive to the immune response, and more T lymphocytes appeared in the TME and were further activated by the PD-1 blockade. In other words, DAC enhanced the anti-tumor effect of the PD-1 blockade and improved the survival rate in CRC.

## DISCUSSION

Tumor immunotherapies such as chimeric antigen receptor T cells and PD-1 blockade have achieved exciting clinical outcomes. However, the overall effective rate of PD-1 blockade in solid tumors is only 20–30%. The inhibitory status of the TME largely impairs the effect of immunotherapy.^29^ The intensity of the immune response in the TME crucially determines the effectiveness of immunotherapy.^3–5,30^ The microenvironment in tumors that are MSI-H/dMMR is immune responsive, which provides evidence and a reference for other strategies. How to change a cold tumor (low immunoreactivity) to a hot tumor (high immunoreactivity) has become a hot topic. Therefore, multiple strategies such as DC vaccines, oncolytic viruses, and radiotherapy have been explored to modulate the TME with the goal of enhancing the anti-tumor effect.^31–35^ For example, radiation-damaged tumor cells not only undergo a so-called immunogenic death that effectively exposes tumor antigens and triggers an antitumor immune response but also activate APCs through the release of damage-associated molecular pattern molecules (DAMPs).^36^ Radiation therapy has the potential to trigger antigen-specific, adaptive immunity, a phenomenon called “in situ” vaccination, which improves the efficiency and survival rate combining with PD-1 blockade or another immunotherapy.^37,38^

In this study, we have demonstrated that low-dose DAC could modify the CT26 cells and the PDX tumor cells by demethylation of the DNA promoter regions, which increased the expression of antigen-processing and antigen-presenting genes and other immune-related genes. In CT26 cells, H2-T10, H2-Q4 and H2-T23 of the mouse MHC complex were significantly upregulated (Figs. 3d and 4), and this was closely related to the presentation of tumor antigens. This is similar to the result reported by Schrump.^39^ Correspondingly, cytokine genes and chemokine genes such as TGFB3 and CXCL1 were activated. These results clearly indicate that the intensity of the immune response in the TME was enhanced, which changed the cold tumor to a hot one.

Furthermore, we established a PDX model of CRC with MSS in immunodeficient mice. DAC was administered, and the characteristics of the tumor cells were profiled. We found that the proteins were significantly modified compared to the control, and immune-related genes such as IL36RN, IL1RN, and NFKBIA were regulated by DAC, which suggested that the immunogenicity of the tumor cell was improved. Consistent with previously reported studies,^40,41^ our results showed a strong correlation between the gene methylation level and gene activation. Therefore, low-dose DAC changed the characteristics of tumor cells to become more immune responsive.

The changes in tumor cells resulted in the re-modulation of the TME, which increased the infiltration of T lymphocytes to the tumor site. Unfortunately, we found that most of the infiltrated T lymphocytes were PD-1 positive (Fig. 6c), which indicated that they would be anergic and dysfunctional. This might be due to the activation of the STAT-related genes,^42,43^ as shown in Fig. 3d. If so, PD-1 blockade might be effective in this context. Above all, none of the genes associated with MMR are mutated in CT26, which demonstrated that the CT26 CRC cells are MMR-proficient or MSS.^44^ Of special importance, we first proved that low-dose DAC could alter the profiles of the CRC cells with MSS, and the combination of low-dose DAC and PD-1 blockade significantly inhibited the tumor growth of CT26 CRC cells and improved the survival rate of the tumor-bearing mice. Our findings strongly suggested that the treatment strategy of the joint application of PD-1 blockade and low-dose DAC would be effective in future clinical trials.

Increasing evidence has accumulated that shows that DAC alone or combined with other epigenetic reagents such as histone deacetylase inhibitors can specifically improve the expression of the testicular cancer antigens NY-ESO-1; MAGE-1, MAGE-2, MAGE-3; and GAGE^39^ in tumor cells but not in normal epithelial cells.^45,46^ Furthermore, the de novo expression of NY-ESO-1 induced by DAC was found to be persistent. Its mRNA and protein could still be detected in some renal cell lines 50–60 days after the DAC treatment.^47^ Our findings also demonstrated that the low-dose DAC could reactivate the silent genes by demethylation of their promoters. The function of these genes was vital for the crosstalk between the tumor and immune system, especially for the immune response that was triggered by the reactivation of antigen-processing and antigen-presenting genes, cytokine-related genes, chemokine-related genes and other immune-related genes. Moreover, DAC not only modified the tumor cells but also promoted the T-cell proliferation in vivo.^48^ This made DAC the ideal pretreatment drug for immunotherapy.^49^

It is known that the efficiency of PD-1 blockade is largely dependent upon the characteristics of tumor mutation burden or MSI-H. High MSI usually results in more mutations and the production of unknown proteins that can be recognized by the immune system. More tumor mutation burden means more infiltrated T lymphocytes, which is similar to the mechanism in our hypothesis. It is essential that enough anergic T lymphocytes are reactivated by the PD-1 blockade to kill the tumor cells. However, one of the key problems of immunotherapy is the low immunogenicity of the tumor and the hypo-immunoreactivity or immune-anergy of the immune system. It will be essential to determine how to improve the tumor immunogenicity such that the tumor can be recognized by the immune system. Notably, a large number of researchers have reported that DNA methylation may be more closely related to cancer than gene mutation. Up to 5% of the known genes in tumor cells have promoters that are abnormally hypermethylated. Most of these genes are known tumor-suppressor genes, which are silenced.^50^ However, DNA analysis of various tumor cells has found that the probability of gene mutation in cancer cells is much lower than expected.^51^ The possibility that antigen genes and immune response-related genes could be reactivated by DAC could provide a novel immunological boost to cancer patients.^52^ Fortunately, the expression of testicular cancer antigens induced by DAC has not been found to occur in normal epithelial cells; therefore, these normal cells are able to escape from the immune attacks. It is promising that DAC would convert a “cold tumor” to a “hot tumor” that would be more responsive to the immunotherapy.

Collectively, we have provided a novel therapeutic application of DAC for CRC patients. DAC-based TME re-modulation could create more suitable conditions for other immunotherapies, for example PD-1 blockade. Strategies that combine low-dose DAC with immunotherapy for CRC patients, especially those with MSS, are promising but need to be confirmed by more clinical evidence.
